# Characteristics of non-cardia gastric cancer with a high serum anti-*Helicobacter pylori* IgG titer and its association with diffuse-type histology

**DOI:** 10.1371/journal.pone.0195264

**Published:** 2018-04-05

**Authors:** Eun Jeong Gong, Ji Young Lee, Suh Eun Bae, Young Soo Park, Kee Don Choi, Ho June Song, Gin Hyug Lee, Hwoon-Yong Jung, Woo Jin Jeong, Gab Jin Cheon, Jeong Hwan Yook, Byung Sik Kim

**Affiliations:** 1 Department of Internal Medicine, Gangneung Asan Hospital, University of Ulsan College of Medicine, Gangneung, Korea; 2 Health Screening and Promotion Center, Asan Medical Center, Seoul, Korea; 3 Department of Gastroenterology, University of Ulsan College of Medicine, Asan Medical Center, Seoul, Korea; 4 Department of Pathology, University of Ulsan College of Medicine, Asan Medical Center, Seoul, Korea; 5 Department of Surgery, University of Ulsan College of Medicine, Asan Medical Center, Seoul, Korea; National Cancer Center, JAPAN

## Abstract

**Background:**

Data on implications of a high positive titer of serum anti-*Helicobacter pylori* antibody on gastric cancer (GC) is limited. This study aimed to investigate the characteristics of GC with a high serum anti-*H*. *pylori* IgG (Hp-IgG) titer, and its association with diffuse-type GC.

**Methods:**

We analyzed clinical and histological characteristics of 917 non-cardia GC patients who underwent gastrectomy. *H*. *pylori* infection was determined serologically by measuring Hp-IgG titer with immunoassay. Seropositive patients were divided into three groups (low-positive, mid-positive, and high-positive) according to the Hp-IgG titer value. Tumors were classified according to the Lauren criteria as diffuse or intestinal types.

**Results:**

The median age of the patients was 59.0 years, and 33.8% were female. The patents were grouped as follows: seronegative, 188 (20.5%); low-positive, 288 (31.4%); mid-positive, 290 (31.6%); and high-positive 151 (16.5%). The high-positive group was significantly younger (median age, 55.0 years), with a higher proportion of female (45.0%) and non-smokers (58.9%). The proportion of diffuse-type GC increased in the order low-, mid-, and high-positive groups (*p*<0.001). In univariate analysis, the factors associated with diffuse-type GC were younger age, female sex, non-smokers, and a high-positive Hp-IgG titer. Younger age, female sex, and non-smokers remained significant on multivariate analysis whereas the high-positive Hp-IgG titer showed only a tendency toward the association (*p* = 0.078).

**Conclusions:**

Non-cardia GC patients with a high Hp-IgG titer have distinct clinicopathologic characteristics. A high-positive Hp-IgG titer should be interpreted together with patients’ age, sex, and smoking status.

## Introduction

Non-cardia gastric cancer (GC) develops with a sequence of events that evolves from atrophic gastritis and intestinal metaplasia to dysplasia and carcinoma [1]. *Helicobacter pylori* infection is the main cause of gastritis, and is a well-known risk factor for GC [2]. Serum anti-*H*. *pylori* IgG antibody (Hp-IgG) test is widely used to determine the presence of *H*. *pylori* infection [3,4] and plays a useful role in risk assessments for the development of GC, particularly in combination with serum pepsinogen [5–7].

A close association has been observed between the histological type of GC and *H*. *pylori* infection status, with the proportion of diffuse-type GC being higher in patients with current infection than in those with past infection [8]. In addition, several studies including our previous study have suggested that subjects with a low Hp-IgG titer are at high risk for intestinal-type GC, whereas those with a high Hp-IgG titer are at increased risk for diffuse-type GC [9–12]. Chronological change in Hp-IgG titers which decrease with the progression of gastric mucosal atrophy may be attributable to the association between past infection or low Hp-IgG titer and intestinal-type GC [8]. Meanwhile, a high Hp-IgG titer may reflect mucosal inflammation resulting from active *H*. *pylori* infection and thus may be related to the development of diffuse-type GC [7,10,11]. However, the association between Hp-IgG titer and the histological type of GC remains unclear because comprehensive analyses including age, sex, and other related factors are limited.

Given this context, our study was aimed to elucidate the characteristics of non-cardia GC with a high-positive Hp-IgG titer.

## Materials and methods

### Subjects

**Fig 1:**
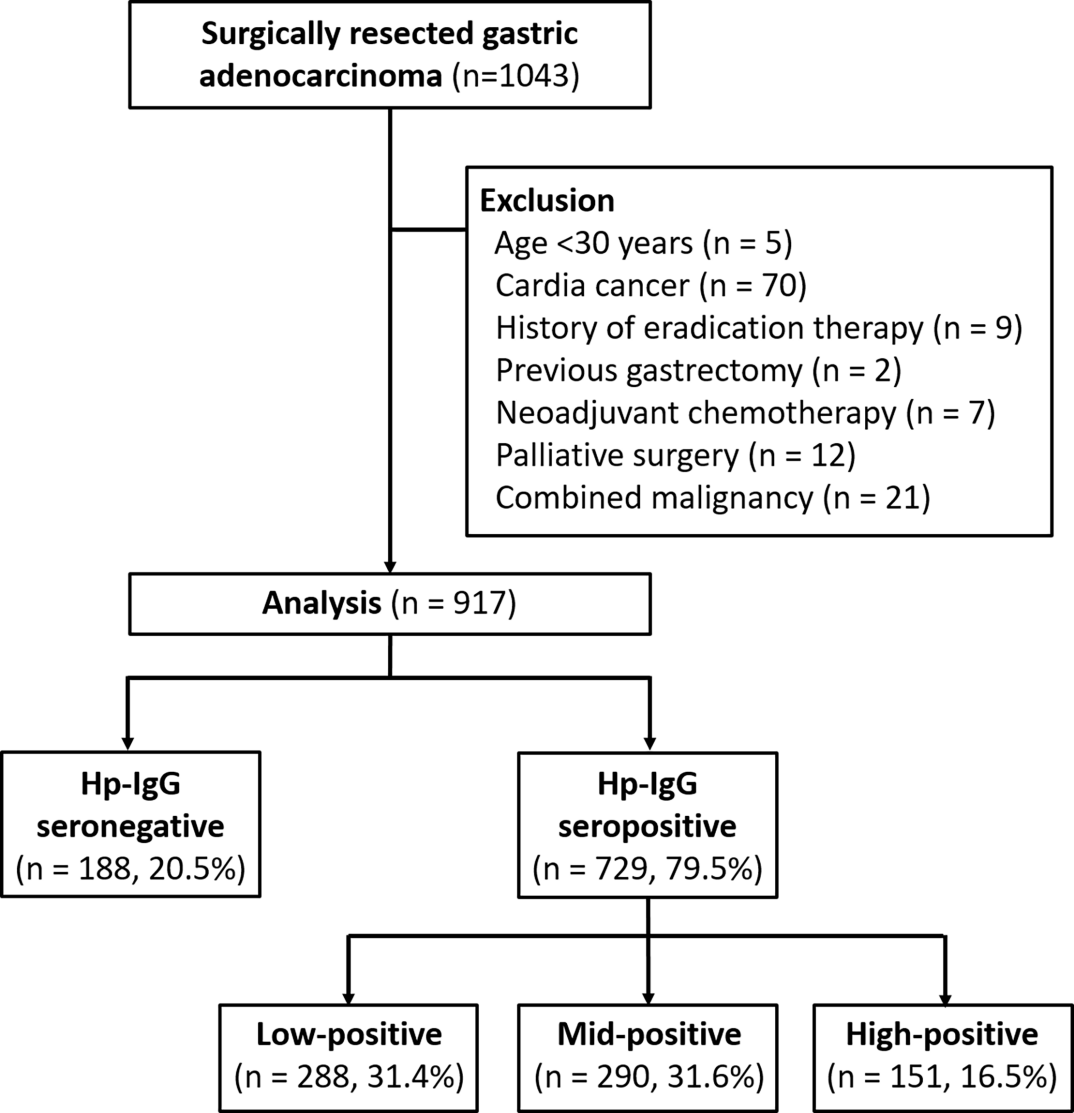
Study patients and their classification into groups according to their serum anti-*Helicobacter pylori* IgG antibody (Hp-IgG) titer.

Data were retrospectively retrieved from the medical records of 1034 patients with non-cardia GC who underwent surgical gastrectomy between April 2012 and March 2014 at Asan Medical Center, Seoul, South Korea. Serum Hp-IgG was measured at the time of GC diagnosis. After applying predetermined exclusion criteria, 126 patients were excluded from the analysis for the following reasons: 5 for being aged under 30 years, 70 for cardia cancer, 9 for history of eradication therapy, 2 for the previous gastrectomy, 7 for neoadjuvant chemotherapy, 12 for palliative surgery, and 21 for combined malignancies. Ultimately, 917 patients with non-cardia GC were included in our analysis (Fig 1). The study protocol was approved by the Institutional Review Board of Asan Medical Center, which confirmed that it accorded with the ethical principles of the Declaration of Helsinki.

### Serum anti-*H*. *pylori* IgG antibody

Blood samples were drawn from each patient and transported to the laboratory, where the serum was separated and tested immediately. After complete clot formation, centrifugation was performed at 3500 rpm for 10 minutes. Serological positivity to *H*. *pylori* infection was determined using a solid-phase, chemiluminescent immunometric assay, commercially available IMMULITE 2000^®^ immunoassay system (Siemens Healthcare Diagnostics Products Ltd., Llanberis, UK)[13]. This test was validated in Korean population, and the sensitivity and specificity were reported to be 97.0% (95% CI 89.6%–99.6%) and 100.0% (95% CI 95.7%–100.0%), respectively [14]. The positive and negative predictive values were 100.0% (95% CI unavailable) and 97.7% (95% CI 93.9–100.0%).

The calibration range of this immunoassay is 0.4–8.0 U/mL, with the Hp-IgG titer classified as follows: negative, 0–1.0 U/mL; positive, 1.1–7.9 U/mL; and high positive, ≥8.0 U/mL. We divided the patients in the H. pylori-positive group into two approximately equal subgroups according to a cutoff value chosen to ensure the same number of cases for each subgroup: the low-positive group (1.1–4.2 U/mL, n = 288) and the mid-positive group (4.3–7.9 U/mL, n = 290) (Fig 1)[9,12].

### Histological classifications

The tumors were classified as diffuse-type or intestinal-type on the basis of Lauren’s criteria [15]. If a tumor exhibited both histologic types, it was categorized as the predominant histologic type. The tumors were also categorized as differentiated or undifferentiated according to the degree of glandular differentiation based on the World Health Organization criteria [16]. Tumor location was specified as upper, middle, or lower third by dividing the stomach with lines that connected the trisected points of the lesser and greater curvatures [17]. Non-cardia GC was defined if the epicenter of the tumor was located more than 2 cm distal to the gastroesophageal junction. Early GC was defined as GC confined to the mucosa or submucosa, irrespective of regional lymph node metastasis [17]. The pathological GC staging followed the guidelines of the seventh edition of the American Joint Committee on Cancer [18].

### Statistical analysis

Baseline variables are presented as number (percentage) or median (interquartile range, IQR). Differences between characteristics were determined using the chi-square test, Fisher’s exact test, the Mann-Whitney *U* test, or the Kruskal Wallis test, as appropriate. Factors associated with diffuse-type histology were assessed using a logistic regression model to calculate odds ratios with the corresponding 95% confidence intervals. SPSS version 23.0 (SPSS Inc., Chicago, IL) was used for all statistical analyses, and a *p*-value <0.05 was considered significant.

## Results

### Comparison of serum anti-*H*. *pylori* IgG negative and positive gastric cancers

The median age of study patients was 59.0 years (IQR, 51.0–69.0 years) and the male-to-female ratio was 1.96:1 (Table 1). Serum Hp-IgG was positive in 729 patients (79.5%); these patients were significantly younger than the Hp-IgG-negative patients (median age, 58.0 and 64.0 years, respectively; *p*<0.001). Other variables, including sex, smoking history, family history of GC, histological classification, and tumor stages, did not differ significantly between Hp-IgG positive and negative groups.

**Table 1:** Comparison between serum anti-*H*. *pylori* IgG (Hp-IgG) negative and positive non-cardia gastric cancers (GC).

	Total (n = 917)	Hp-IgG negativeGC (n = 188)	Hp-IgG positiveGC (n = 729)	*P-*value
Age, years (IQR)	59.0 (51.0–69.0)	64.0 (55.3–72.0)	58.0 (49.0–67.0)	<0.001
Sex, female	310 (33.8)	58 (30.9)	252 (34.6)	0.337
Smokers	487 (53.1)	102 (54.3)	385 (52.8)	0.724
Family history of GC*	200 (21.8)	44 (23.4)	156 (21.4)	0.553
Tumor location				0.732
Upper	105 (11.5)	22 (11.7)	83 (11.4)	
Middle	305 (33.3)	58 (30.9)	247 (33.9)	
Lower	507 (55.3)	108 (57.4)	399 (54.7)	
Tumor size, mm (IQR)	30.0 (20.0–47.0)	30.0 (21.0–51.5)	30.0 (20.0–46.5)	0.703
Gross tumor morphology				0.866
Early GC	663 (72.3)	135 (71.8)	528 (72.4)	
Advanced GC	254 (27.7)	53 (28.2)	201 (27.6)	
Glandular differentiation				0.105
Differentiated	372 (40.6)	86 (45.7)	286 (39.2)	
Undifferentiated	545 (59.4)	102 (54.3)	443 (60.9)	
Lauren classification				0.098
Diffuse-type	493 (53.8)	91 (48.4)	402 (55.1)	
Intestinal-type	424 (46.2)	97 (51.6)	327 (44.9)	
GC stage				0.442
Stage I	697 (76.0)	138 (73.4)	559 (76.7)	
Stage II	117 (12.8)	24 (12.8)	93 (12.8)	
Stage III	103 (11.2)	26 (13.8)	77 (10.6)	

Data are presented as the number of patients (%) or the median (interquartile range, IQR).

*First-degree relatives.

### Comparison of gastric cancer groups according to serum anti-*H*. *pylori* IgG titer

There were significant differences in age, sex, and smoking history among the serum Hp-IgG titer groups (Table 2). The patients in the high-positive group were the youngest, with the highest proportions of female and non-smokers. The proportions of undifferentiated and diffuse-type GC both increased in the order low-, mid-, and high-positive groups (*p*<0.001). Diffuse-type GC accounts for 68.9% of the patients in the high-positive group.

**Table 2:** Comparisons between the serum anti-*H*. *pylori* IgG (Hp-IgG) titer groups.

	Hp-IgG negative GC(n = 188)	Hp-IgG positive GC (n = 729)			*P-*value
		Low-positive(n = 288)	Mid-positive(n = 290)	High-positive(n = 151)	
Age, years (IQR)	64.0(55.3–72.0)	59.0(52.0–70.0)	56.0(48.0–66.0)	55.0(48.0–65.0)	<0.001
Sex, female	58 (30.9)	90 (31.3)	94 (32.4)	68 (45.0)	0.016
Smokers	102 (54.3)	155 (53.8)	168 (57.9)	62 (41.1)	0.009
Family history of GC*	44 (23.4)	64 (22.2)	64 (22.1)	28 (18.5)	0.737
Tumor location					0.336
Upper	22 (11.7)	23 (8.0)	40 (13.8)	20 (13.2)	
Middle	58 (30.9)	105 (36.5)	92 (31.7)	50 (33.1)	
Lower	108 (57.4)	160 (55.6)	158 (54.5)	81 (53.6)	
Tumor size, mm (IQR)	30.0 (21.0–50.5)	33.0 (23.0–53.0)	29.0 (20.0–44.0)	30.0 (20.0–45.0)	0.047
Gross tumor morphology					0.126
Early GC	135 (71.8)	198 (68.8)	210 (72.4)	120 (79.5)	
Advanced GC	53 (28.2)	90 (31.3)	80 (27.6)	31 (20.5)	
Glandular differentiation					<0.001
Differentiated	86 (45.7)	133 (46.2)	113 (39.0)	40 (26.5)	
Undifferentiated	102 (54.3)	155 (53.8)	177 (61.0)	111 (73.5)	
Lauren classification					<0.001
Diffuse-type	91 (48.4)	140 (48.6)	158 (54.5)	104 (68.9)	
Intestinal-type	97 (51.6)	148 (51.4)	132 (45.5)	47 (31.1)	
GC stage					0.072
Stage I	138 (73.4)	208 (72.2)	226 (77.9)	125 (82.8)	
Stage II	24 (12.8)	38 (13.2)	37 (12.8)	18 (11.9)	
Stage III	26 (13.8)	42 (14.6)	27 (9.3)	8 (5.3)	

Data are presented as the number of patients (%) or the median (interquartile range, IQR).

GC, non-cardia gastric cancer

*First-degree relatives.

### Diffuse-type gastric cancer in each anti-*H*. *pylori* IgG titer group by age, sex, and smoking status

**Fig 2:**
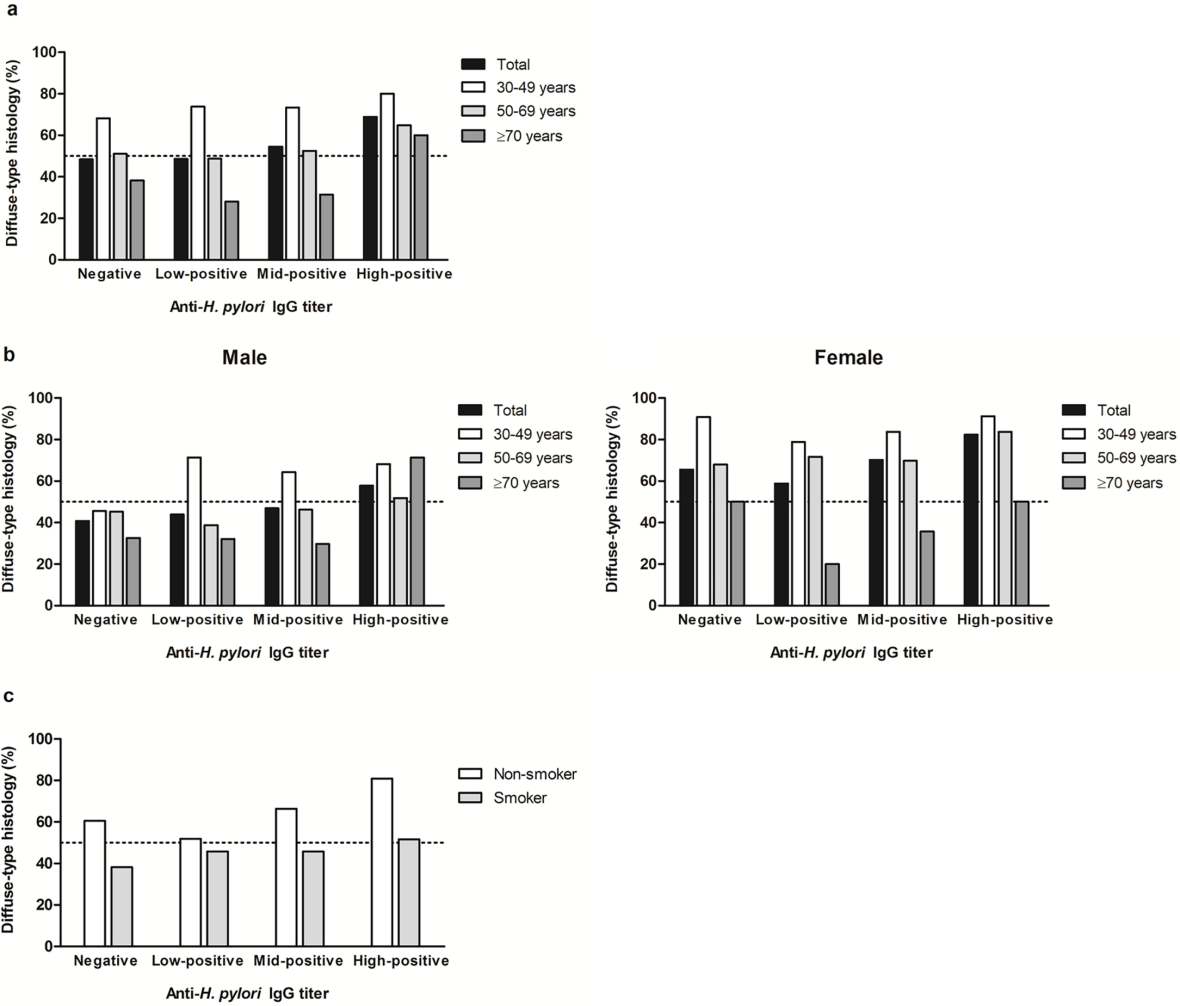
The proportion of diffuse-type non-cardia gastric cancer (GC) in each Hp-IgG titer group according to age, sex, and smoking status. (a) Age: the proportion of diffuse-type GC was highest in the patients aged 30–49 years and decreased with advancing age (*p*<0.001). (b) Age and sex: this difference between age groups was significant in all Hp-IgG titer groups in female. (c) Smoking status: Among the non-smokers, the proportion of diffuse-type GC increased in the higher Hp-IgG titer groups (*p*<0.001).

Fig 2 shows the proportion of diffuse-type GC in each Hp-IgG titer group subdivided by age, sex, and smoking status. Diffuse-type GC was predominant in the high-positive group regardless of age. In all of the Hp-IgG titer groups, the proportion of diffuse-type GC was highest in the patients aged 30–49 years and decreased with advancing age (*p*<0.001; Fig 2A). This tendency was prominent in all Hp-IgG titer groups in female GC patients (Fig 2B). Indeed, the proportion of diffuse-type GC was 91.3% (21/23) in female aged 30–49 years with high-positive Hp-IgG titer. Among the non-smokers, the proportion of diffuse-type GC increased with the higher Hp-IgG titer (*p*<0.001; Fig 2C).

### Association between serum anti-*H*. *pylori* IgG titer and diffuse-type gastric cancer

Logistic regression analysis was performed to evaluate the association between the serum Hp-IgG titer and diffuse-type GC (Table 3). Univariate analysis showed that younger age, female sex, non-smokers, and being in the high-positive group were factors associated with diffuse-type GC. Younger age, female sex, and non-smokers remained significant on multivariate analysis. Being in the high-positive Hp-IgG group showed only a tendency toward the association (*p* = 0.078).

**Table 3:** Factors associated with diffuse-type histology in non-cardia gastric cancer.

	Univariate analysis		Multivariate analysis	
	Odds ratio (95% CI)	*P-*value	Odds ratio (95% CI)	*P-*value
Age, years				
30–49	Reference		Reference	
50–69	0.393 (0.274–0.562)	<0.001	0.427 (0.295–0.618)	<0.001
≥70	0.181 (0.118–0.276)	<0.001	0.188 (0.120–0.293)	<0.001
Female	2.564 (1.922–3.421)	<0.001	1.824 (1.220–2.728)	0.003
Non-smoker	2.149 (1.648–2.803)	<0.001	1.516 (1.041–2.207)	0.030
Anti-*H*. *pylori* IgG titer group				
Negative	Reference		Reference	
Low-positive	1.008 (0.698–1.456)	0.965	0.847 (0.573–1.253)	0.407
Mid-positive	1.276 (0.883–1.843)	0.194	0.994 (0.669–1.477)	0.975
High-positive	2.359 (1.507–3.691)	<0.001	1.538 (0.953–2.481)	0.078

CI, confidence interval

## Discussion

In this study, GC patients with a high-positive Hp-IgG titer exhibited distinct clinicopathological characteristics. The high-positive Hp-IgG titer group was younger and consisted of higher proportions of female and non-smokers than the other groups. The proportion of diffuse-type GC increased in the order of low-, mid-, and high-positive groups. When considering age, sex, and smoking status, the high-positive Hp-IgG titer showed only a tendency toward the association with diffuse-type GC histology.

Several studies have investigated the clinicopathologic features of GC according to *H*. *pylori* infection status [8,19–22]. In one study of resectable GC, *H*. *pylori* seropositivity was associated only with a tumor location in the lower third of the stomach [19]. Other studies evaluating the association between *H*. *pylori* infection and histological type of GC found no differences in the proportions of diffuse- and intestinal-type GC between seropositive and seronegative patients [20–22]. Conversely, a recent study reported clinicopathologic characteristics of GC by classifying GC patients into three groups according to whether they had a current *H*. *pylori* infection, past infection serologically, or a past infection only identified histologically [8]. The patients with a past *H*. *pylori* infection were older and with a greater predominance of male than those with a current infection. The proportion of diffuse-type GC was much higher in the patients who were currently infected with *H*. *pylori* than in those infected in the past. Further, patients with a serologic past infection had a higher proportion of diffuse-type GC compared with those only with a histological evidence of past infection (38.2% vs. 19.8%).

Previously, we reported that 90.7% of diffuse-type early GC patients were Hp-IgG positive (vs. 75.9% of intestinal-type GC), and that the proportion of diffuse-type early GC increased significantly with increasing Hp-IgG titers [12]. These results have led to a speculation that a quantitative approach using Hp-IgG titer could be useful for the further characterization of GC patients. In the present study, we categorized seropositive patients into subgroups using Hp-IgG titer values. Our results showed that the median age of patients decreased in the order of negative, low-positive, mid-positive, and high-positive Hp-IgG titer groups. In addition, the proportions of female and diffuse-type GC increased from the low- to high-positive groups. Notably, these differences were not significant when the patients were categorized dichotomously into seronegative and seropositive groups.

Several studies suggested an association between Hp-IgG titer and the histological type of GC [7,9]. The Hp-IgG titer is associated with the severity of gastritis [10,23,24], and *H*. *pylori*-induced inflammation activity has been shown to be well correlated with serum Hp-IgG or pepsinogen II levels [3,4]. A previous study regarding the Hp-IgG titer and the risk of GC demonstrated that subjects with a low Hp-IgG titer in the presence of mucosal atrophy were at high risk for intestinal-type GC, whereas those with a high Hp-IgG titer were at high risk for diffuse-type GC [9]. This association between higher Hp-IgG titer and the development of diffuse-type GC was significant in the absence of gastric atrophy [10,11].

In this study, *H*. *pylori* infection was defined solely by serum Hp-IgG titers. The negative Hp-IgG titer group may consist of heterogeneous patients, including those of truly infection negative, those with advanced atrophic gastritis, or those with previous *H*. *pylori* infection successfully eradicated [7]. The proportion of Hp-IgG negative GC was 20.5%, which is relatively higher than that of reported in previous Korean studies [8,20–22]. Long-term *H*. *pylori* infection induces mucosal atrophy, leading to hypoacidity and subsequent hypergastrinemia that predispose to GC development [25]. C. *H*. *H*omprehensive analysis with serum gastrin may add useful value to the characterization of *H*. *pylori*-induced GC [26]. Nevertheless, our present study with Hp-IgG quantitative approach may also help the understanding of clinicopathologic characteristics of non-cardia GC, particularly for those with a high-positive Hp-IgG titer.

Younger age and female sex is associated with diffuse-type GC [15]. In this study, the proportion of diffuse-type GC was highest in young patients aged 30–49 years, decreasing with advancing age; the ratio of diffuse- to intestinal-type GC was 2.91 in patients aged 30–49 years and 0.53 in those aged ≥70 years. Of note, even in those aged ≥70 years, the diffuse-type GC was also often in the high-positive Hp-IgG group. This tendency was prominent in female GC patients with high-positive Hp-IgG titer.

Regarding the influence of smoking on histological type of GC, few studies have demonstrated that smoking was associated with an increased risk of differentiated or intestinal-type GC [27,28]. In our previous study, we also found that more patients with intestinal-type GC had histories of smoking than those with diffuse-type GC [12]. This could be partly explained by the hypothesis that the intestinal-type GC is more likely related to environmental factors than the diffuse-type GC [29]. In the present study, non-smokers were associated with an increased risk of diffuse-type GC. In addition, the proportion of diffuse-type GC increased with the higher Hp-IgG titer groups only among the non-smokers, suggesting complex relationship between smoking, Hp-IgG titer, and GC histology. In a multivariate analysis of these factors, including age, sex, and smoking status, we observed only a marginally significant association was observed between the high-positive Hp-IgG titer group and diffuse-type GC. Therefore, the association between a high-positive Hp-IgG titer and diffuse-type GC should be interpreted together with patients’ age, sex, and smoking status, rather than as an independent factor.

Our study had some limitations inherent to its retrospective design and the use of surgical data collected in a single center. This study could not evaluate the implication of Hp-IgG titer in a whole range of GC patients, including those with GC eligible for endoscopic resection or those with far advanced unresectable GC. In addition, other confounding factors such as socioeconomic status, alcohol consumption or dietary habits were not fully assessed. Although we excluded patients being aged under 30 years, there might be a rare possibility of inclusion of *H*. *pylori*-unrelated GC in the Hp-IgG negative GC group.

In conclusion, non-cardia GC patients with a high Hp-IgG titer have distinct clinicopathologic characteristics. Given the trend toward a positive association with diffuse-type GC in multivariate analysis, a high-positive Hp-IgG titer should be interpreted together with patients’ age, sex, and smoking status.

## Supporting information

S1 File Data. Data of the study population.
(SAV)
